# Epidemiology of Visceral Leishmaniasis in Algeria: An Update

**DOI:** 10.1371/journal.pone.0099207

**Published:** 2014-06-20

**Authors:** Amel Adel, Amel Boughoufalah, Claude Saegerman, Redgi De Deken, Zahida Bouchene, Abdelkrim Soukehal, Dirk Berkvens, Marleen Boelaert

**Affiliations:** 1 Institute of Veterinary Sciences, University Saad Dahlab, Blida, Algeria; 2 Department of Infectious and Parasitic Diseases, Epidemiology and Risk Analysis Applied to Veterinary Sciences, Faculty of Veterinary Medicine, University of Liège, Liège, Belgium; 3 Department of Biomedical Sciences, Institute of Tropical Medicine, Antwerp, Belgium; 4 Department of Health Information, National Institute of Public Health, Algiers, Algeria; 5 University Hospital of Beni Messous, Algiers, Algeria; 6 Department of Public Health, Institute of Tropical Medicine, Antwerp, Belgium; Royal Tropical Institute, Netherlands

## Abstract

Visceral leishmaniasis (VL), a zoonotic disease caused by *Leishmania infantum*, is endemic in Algeria. This report describes a retrospective epidemiological study conducted on human VL to document the epidemiological profile at national level. All human VL cases notified by the National Institute of Public Health between 1998 and 2008 were investigated. In parallel all VL cases admitted to the university hospitals of Algiers were surveyed to estimate the underreporting ratio. Fifteen hundred and sixty-two human VL cases were reported in Algeria between 1998–2008 with an average annual reported incidence rate of 0.45 cases per 100,000 inhabitants, of which 81.42% were in the age range of 0–4 years. Cases were detected year-round, with a peak notification in May and June. One hundred and seventy patients were admitted to the university hospitals in Algiers in the same period, of which less than one in ten had been officially notified. Splenomegaly, fever, pallor and pancytopenia were the main clinical and laboratory features. Meglumine antimoniate was the first-line therapy for paediatric VL whereas the conventional amphotericin B was used for adult patients. Visceral leishmaniasis in Algeria shows the epidemiological profile of a paediatric disease with a decrease of the annual reported incidence rate. However, vigilance is required because of huge underreporting and an apparent propagation towards the south.

## Introduction

Visceral leishmaniasis (VL), a vector-born parasitic disease causing febrile splenomegaly, pancytopenia and wasting, has been reported in Algeria since the beginning of the 20th century [1]. In the Mediterranean basin, VL is caused by *Leishmania infantum* and it is a zoonotic disease with dogs considered to be the main reservoir of the parasite [2], [3]. In Algeria, the sand fly vectors are *Phlebotomus perniciosus* and *P. longicuspis* [4], [5]. The geographical distribution of VL covers all the humid and sub-humid regions in the north of the country [6]. The disease affects mainly children under 5 years of age, most of them belonging to poor families residing in rural areas [7]–[9].

**Figure 1:**
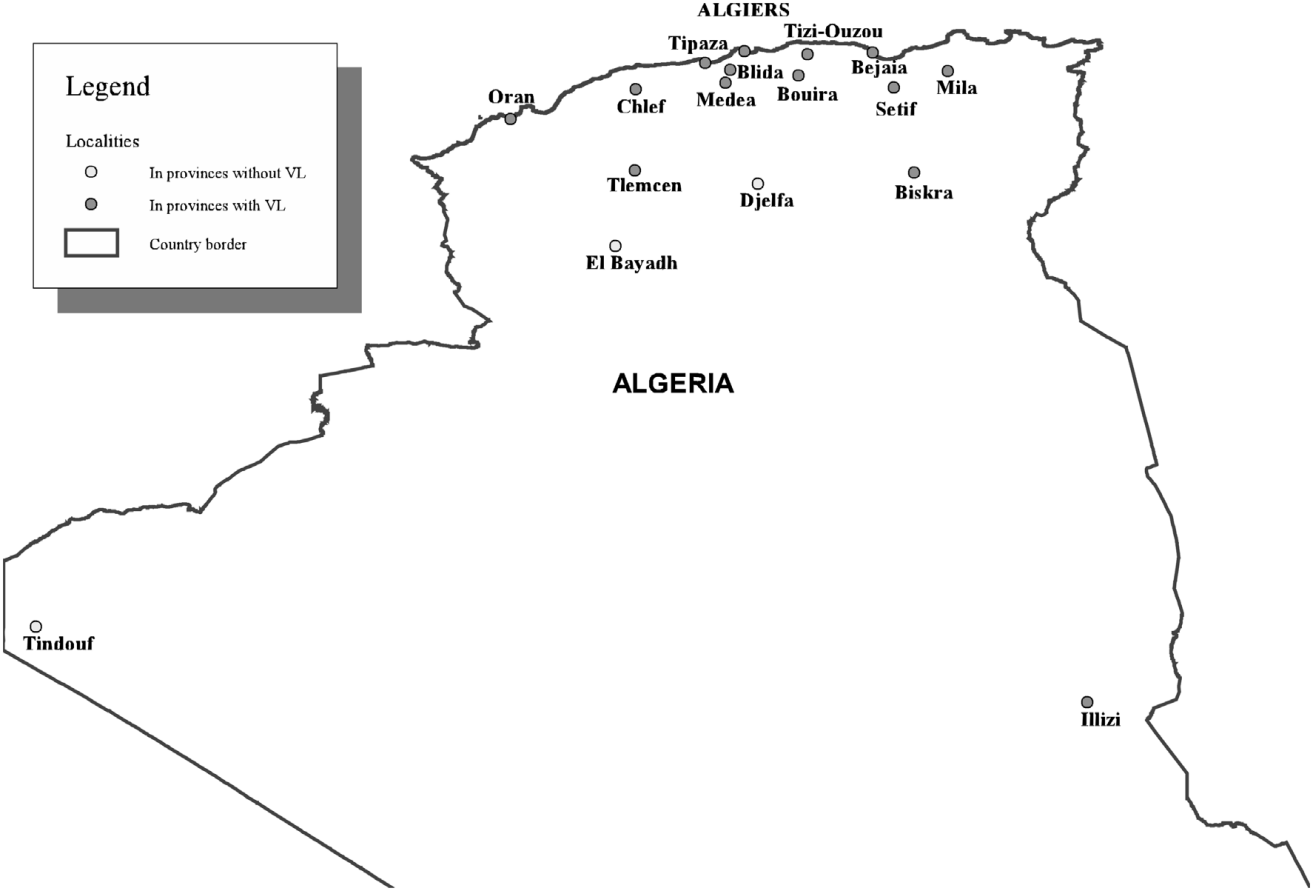
Algeria: localities referred to in the text.

Leishmaniasis is a re-emerging disease in Algeria and seems to spread because of a combination of factors: environmental changes as well as factors related to the immune status of the host and drug resistance [10], [11]. Several epidemiological surveys on VL have been conducted between 1972 and 1990 in the main Algerian hospitals [7], [8], [12]. The most recent national report mentions 1121 cases over a six-year period (1985–1990) contrasting with 721 cases reported during the previous 9 years (1975–1984). It showed an extension of VL from the old foci in Kabylie (Tizi-Ouzou, Bejaïa) to the centre (Blida, Chlef, Medea, Tipaza) and the north-eastern part of northern Algeria, with scattered cases occurring in the West (Oran, Tlemcen, Figure 1). The average annual incidence rate doubled over those periods reaching 0.73 per 100,000 inhabitants per year by 1990. Notification of VL is compulsory in Algeria since 1990. Globally, the World Health Organisation considers that under-reporting is substantial [13], although specifically for Algeria, it was recently estimated as mild (1.2–1.8 fold) [14]. The main objective of the present study is to portray the epidemiological profile of VL in Algeria between 1998 and 2008.

## Materials and Methods

Algeria is the largest country of Africa and it consists of four ecological zones of unequal size. The northern Tell region has a Mediterranean climate, characterised by warm, dry summers and cool, wet winters. The total annual rainfall ranges from 400 to 1000 mm. Natural forests are concentrated in a 100–200 km wide belt in the north of the country [15]. The north-western part of the country is more arid (rainfall 200–400 mm). The climate becomes progressively drier when moving south. The Sahara desert is an arid, windy area with large daily temperature differences and a total annual rainfall below 130 mm. Lastly, the Hoggar Massif (7% of the country) lies south of the Sahara. It is a succession of high altitude desert areas, rising in terraces to an altitude of 2900 m. Administratively, Algeria is divided into 48 provinces. In 2008, 66% of the Algerian population lived in urban areas (National Bureau of Statistics, 2010).

### Data Collection

We compiled all the data on official case notifications of VL in Algeria between 1998 and 2008. Our main data source was the epidemiological surveillance database of the National Institute of Public Health (INSP). The INSP data are the result of passive monitoring, based on the notifications registered by the epidemiological services of all health sectors of the entire Algerian territory (local public health facilities, public hospitals, university hospitals). Private physicians are held to notify the VL cases to the epidemiological service to which they are attached. This database contains anonymous patient data including gender, age, residence and time of hospitalisation of VL patients. To estimate the degree of underreporting, we carried out a survey in 5 university hospitals (UH) of Algiers, the capital city. It should be noted that the UH of Algiers receive patients from all over the country.

A standard form was used to extract epidemiological data from the patient files of all those admitted with a VL final diagnosis to the departments of pediatrics, internal medicine and infectious diseases between January 1998 to March 2009. The two data sources were linked on the basis of date of admission/notification, residence, sex and age.

### Data Analysis

Stata/MP 12.1 [16] was used for the statistical analysis. We computed standardised incidence ratios (SIR) to allow comparisons between provinces taking the national average incidence rate as a reference. The chi-squared test for trend (Mantel-Haenszel) was used to assess time-evolution of VL. A linear correlation analysis of incidence rate and urban population rate (Ministry of Housing and Urban Development, 2010) was performed. The human VL incidence and the urban population rate were plotted on a map with Arcview 3.2 (ESRI, Redlands, California). We used the website http://www.gadm.org/countryres to download the administrative regions of Algeria and we obtained the georeferenced data on the cities of Algeria from http://dateandtime.info.

### Ethical Aspects

Clearance for the study was obtained from the Ministry of Health (Direction of prevention) of Algeria and from the hospitals included in the study. Ethical clearance was obtained from the Institutional Review Board of the Institute of Tropical Medicine, Antwerp. All data handled in this study were treated with due respect for confidentiality and fully and untraceably anonymised by the first author (AA). For the hospital survey the data was extracted from the patient records by the clinician in charge onto an individual questionnaire format, without copying any personal identifiers that would allow identification of individual patients. The main investigator of this study (AA) collected the questionnaires from the clinicians and entered the data in a single database.

## Results

One thousand five hundred and sixty-two (1562) VL cases were reported between 1998 and 2008 from the 48 provinces of Algeria to the INSP (Table 1). The average number of cases in Algeria was 142 cases per year and the average annual incidence rate was 0.45 VL cases per 100,000 inhabitants. One thousand two hundred fifty eight VL cases were between 0–4 years old (81.42%) with an average annual incidence rate of 3.32 cases per 100.000 inhabitants in this group. The male: female ratio was 1.24.

**Table 1:** Provinces of Algeria with the corresponding number of cases and percentage of cases between 1998 and 2008.

Province	98	99	00	01	02	03	04	05	06	07	08	Tot.	Perc.†
Adrar	1	0	0	2	1	0	0	0	0	1	0	5	0.32
Ain defla	7	4	6	1	2	0	3	3	3	1	0	30	1.92
Ain temouchent	3	1	0	0	0	0	1	0	0	2	0	7	0.45
Alger	3	0	1	0	4	0	1	1	4	4	0	18	1.15
Annaba	0	2	4	2	1	0	0	0	1	0	0	10	0.64
Batna	8	3	6	3	3	7	9	8	2	1	1	51	3.27
Bechar	0	1	0	0	0	0	0	0	0	0	0	1	0.06
Bejaia	60	15	39	13	2	11	11	13	6	4	9	183	11.72
Biskra	14	12	11	14	19	15	5	2	10	9	4	115	7.36
Blida	4	0	2	2	0	4	3	3	3	0	0	21	1.34
Bordj bouarreridj	13	1	8	8	4	3	5	5	3	8	0	50	3.2
Bouira	18	7	24	1	1	5	7	10	1	10	6	90	5.76
Boumerdes	5	4	4	3	0	0	7	7	0	0	1	31	1.98
Chlef	0	2	2	1	2	1	0	1	2	3	2	16	1.02
Constantine	1	3	3	1	2	9	5	3	5	2	0	34	2.18
Djelfa	0	0	0	0	0	0	0	0	0	0	0	0	0
El bayadh	0	0	0	0	0	0	0	0	0	0	0	0	0
El oued	0	0	0	0	3	3	0	0	1	2	1	10	0.64
El tarf	0	1	0	1	0	1	0	0	0	0	0	3	0.19
Ghardaia	0	0	6	0	0	8	0	1	0	0	0	15	0.96
Guelma	4	0	5	1	2	0	3	2	2	3	2	24	1.54
Illizi	0	0	0	2	1	0	5	2	1	1	0	12	0.77
Jijel	6	2	6	6	0	0	2	1	2	2	5	32	2.05
Khenchela	1	2	0	0	0	1	1	0	0	0	2	7	0.45
Laghouat	0	0	0	0	0	0	0	0	2	0	0	2	0.13
Mascara	0	0	0	0	0	0	0	0	0	1	0	1	0.06
Medea	11	5	10	6	6	4	4	7	4	4	3	64	4.1
Mila	51	32	30	30	17	21	21	11	14	3	9	239	15.3
Mostaganem	1	0	3	2	1	1	0	0	0	0	0	8	0.51
Msila	3	3	5	1	2	0	1	0	1	0	1	17	1.09
Naama	0	0	0	0	0	0	0	1	1	0	0	2	0.13
Oran	7	5	0	6	7	9	3	1	0	1	0	39	2.5
Ouargla	0	0	0	0	0	0	4	1	1	1	0	7	0.45
Oum el bouaghi	0	3	1	3	4	1	0	1	1	6	2	22	1.41
Relizane	1	0	0	0	0	0	1	0	0	2	5	9	0.58
Saida	0	1	0	0	0	0	1	0	1	0	0	3	0.19
Setif	33	19	21	8	8	9	9	12	9	6	13	147	9.41
Sidi bel abbes	0	0	0	0	0	0	0	1	0	1	0	2	0.13
Skikda	2	2	1	0	4	1	1	3	1	0	2	14	0.9
Souk ahras	0	0	3	0	0	2	2	6	3	7	2	25	1.6
Tamanrasset	5	3	1	2	1	0	0	0	1	1	2	16	1.02
Tebessa	1	0	0	0	0	1	1	1	3	3	2	12	0.77
Tiaret	1	0	0	0	2	0	1	0	1	0	1	6	0.38
Tindouf	0	0	0	0	0	0	0	0	0	0	0	0	0
Tipaza	5	2	5	1	2	0	3	1	2	3	2	26	1.66
Tissemsilt	1	1	1	1	0	5	3	0	0	7	3	22	1.41
Tizi ouzou	38	14	20	6	4	8	7	4	0	5	0	106	6.79
Tlemcen	0	1	0	0	1	1	0	0	2	0	0	5	0.32
Total	308	151	220	127	106	131	130	112	93	104	80	1562	

Tot. = total; Perc. = percentage.

† percentage of cases reported between 1998 and 2008.

**Figure 2:**
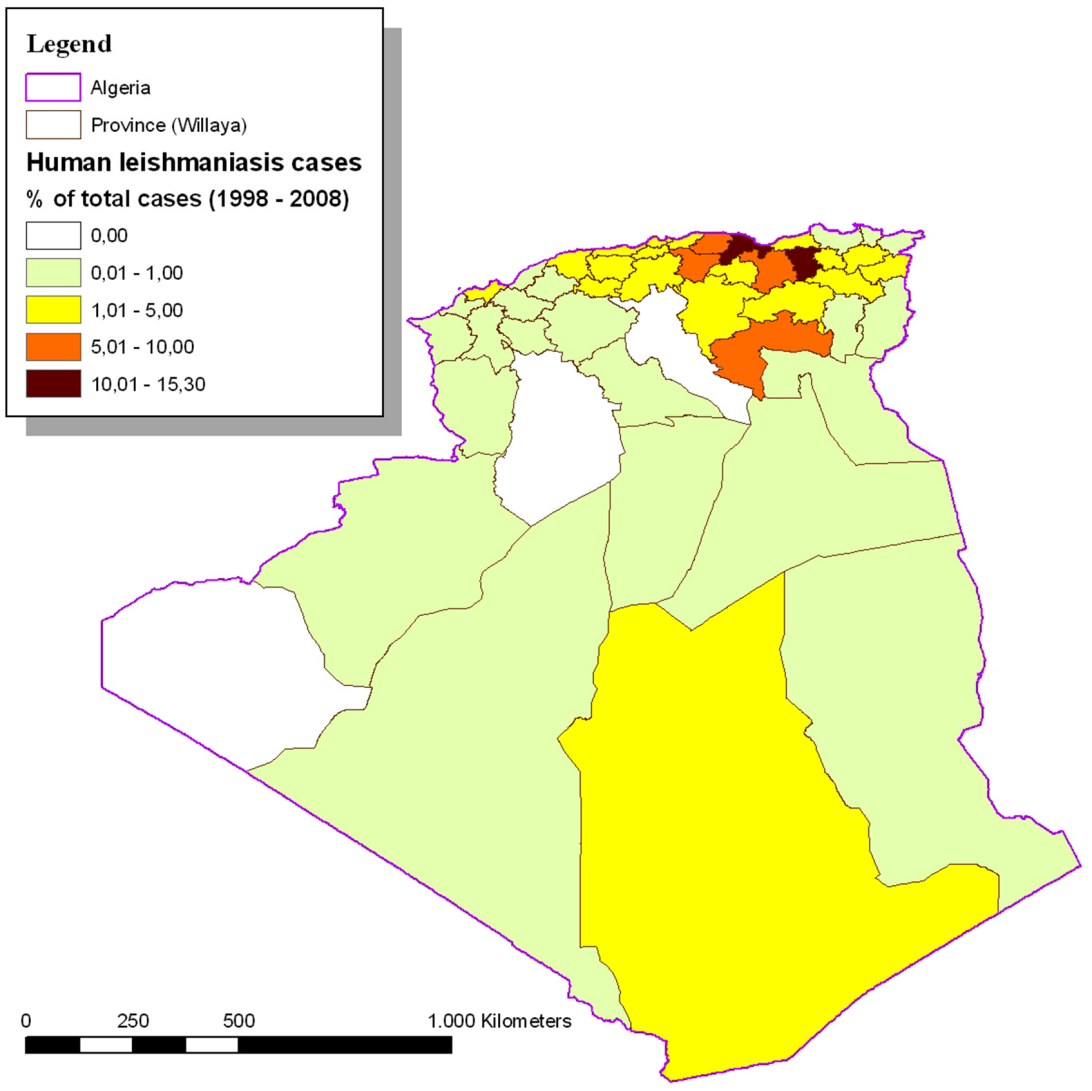
Human visceral leishmaniasis cases in Algeria (percentage of total cases) (1998–2008; N = 1562).

**Figure 3:**
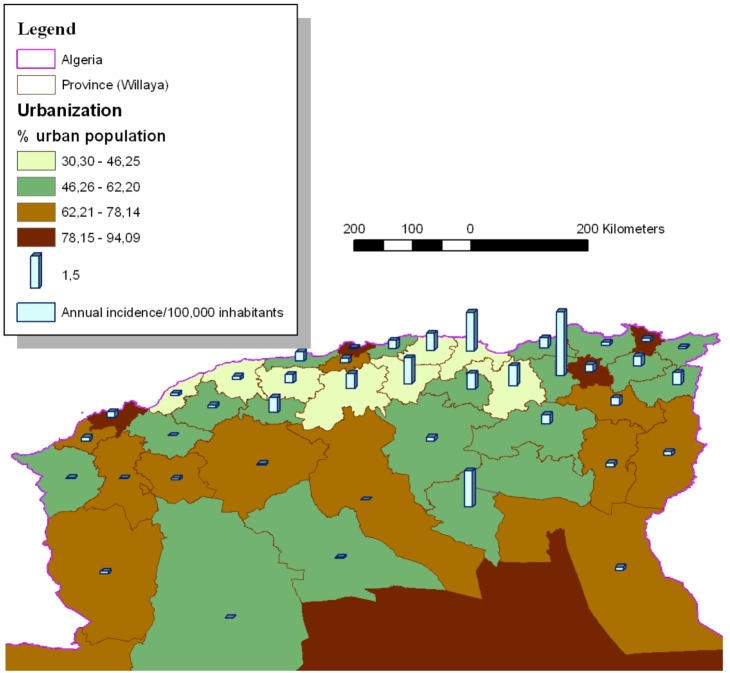
Distribution of human visceral leishmaniasis in North of Algeria, in function of the % of urban population (1998–2008; N = 1562).

**Figure 4:**
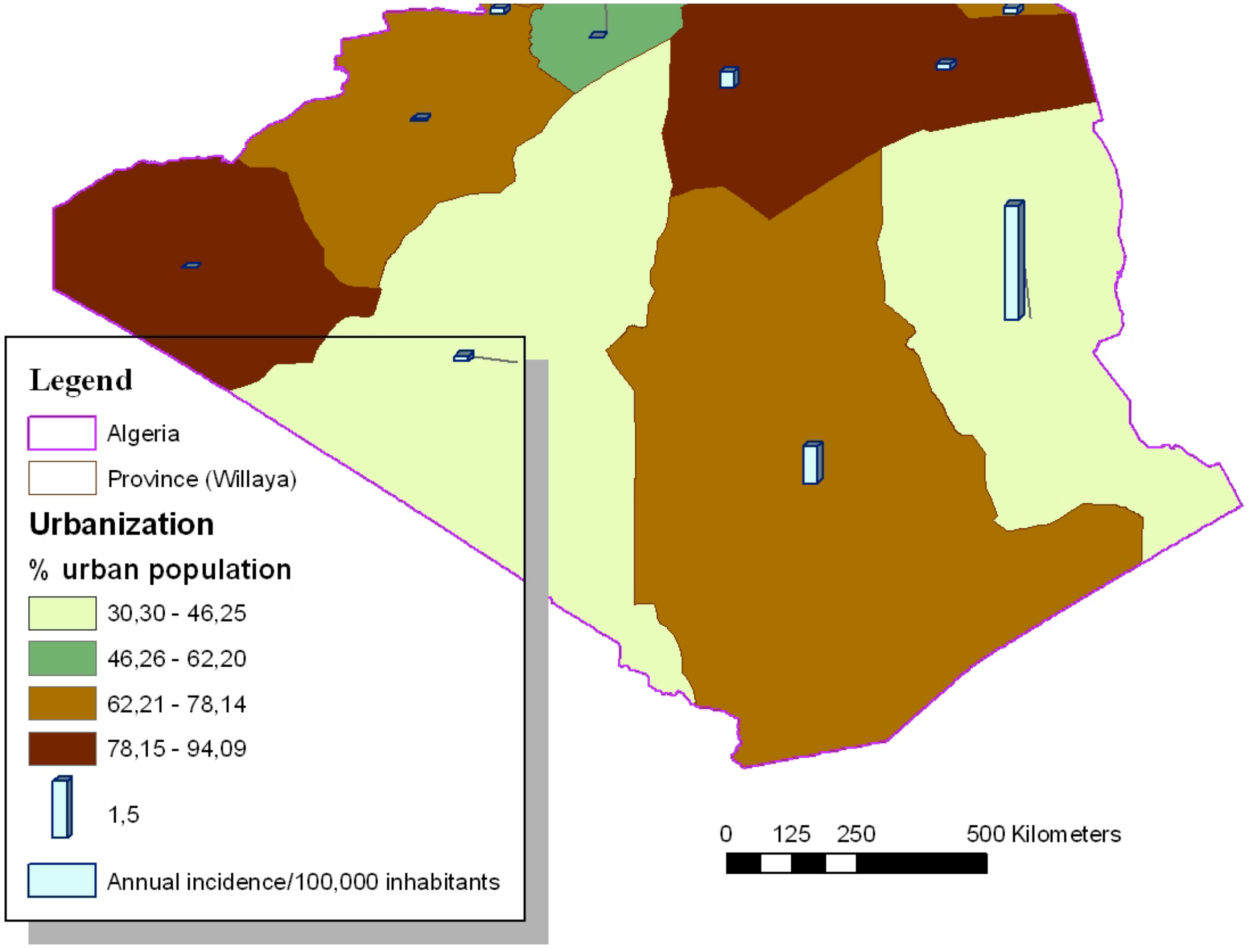
Distribution of human visceral leishmaniasis in South of Algeria, in function of the % of urban population (1998–2008; N = 1562).

One thousand two hundred seventy four of the VL cases (81.56%) came from provinces located in the central and eastern parts of northern Algeria. Mila (239), Bejaia (183), Setif (147), Biskra (115) and Tizi-Ouzou (106) reported the highest number of cases. Table 2 reports the average VL annual incidence rates per 100,000 inhabitants by province with the corresponding SIR. Mila, Bejaia, Illizi, Biskra, Bouira, Tizi-Ouzou and Setif appeared to be above the national average annual incidence rate (Figure 2). The Pearson correlation coefficient between the incidence rate and the urban population rate was −0.34 with a p value of 0.0176 (Figure 3 and Figure 4). In addition to the rural environment, the presence of VL cases could be linked to the proximity of natural parks (Tizi-ouzou and Bouira with Djurdjura park, Bejaia with Gouraya park, Blida with Chrea park, Mila with Taza national park and Illizi with Tassili NAjjer park).

**Table 2:** The average visceral leishmaniasis annual incidence rates per 100,000 inhabitants by province with the corresponding Standardised Incidence Rate (SIR).

Wilaya	Incidence†	SIR	IC 95%
Adrar	0.14	0.03	[0.00; 0.05]
Ain defla	0.39	0.95	[0.61; 1.29]
Ain temouchent	0.18	0.39	[0.10; 0.68]
Alger	0.06	0.16	[0.03; 0.29]
Annaba	0.15	0.47	[0.18; 0.76]
Batna	0.45	1.06	[0.77; 1.35]
Bechar	0.04	0.09	[–0.09; 0.28]
Bejaia	1.8	5.33	[4.56; 6.10]
Biskra	1.7	3.54	[2.90; 4.19]
Blida	0.22	0.62	[0.35; 0.88]
Bordj bouarreridj	0.77	1.77	[1.28; 2.26]
Bouira	1.21	3.1	[2.46; 3.75]
Boumerdes	0.4	0.99	[0.64; 1.33]
Chlef	0.16	0.39	[0.20; 0.59]
Constantine	0.35	1.01	[0.67; 1.34]
Djelfa	0	0	
El bayadh	0	0	
El oued	0.17	0.33	[0.12; 0.53]
El tarf	0.07	0.21	[–0.03; 0.44]
Ghardaia	0.43	0.92	[0.45; 1.38]
Guelma	0.47	1.29	[0.78; 1.81]
Illizi	3.02	3.72	[1.62; 5.83]
Jijel	0.47	1.19	[0.78; 1.60]
Khenchela	0.18	0.43	[0.11; 0.75]
Laghouat	0.05	0.12	[–0.05; 0.28]
Mascara	0.01	0.03	[–0.03; 0.10]
Medea	0.69	1.72	[1.30; 2.15]
Mila	3	7.24	[6.32; 8.15]
Mostaganem	0.11	0.28	[0.09; 0.48]
Msila	0.18	0.39	[0.21; 0.58]
Naama	0.13	0.33	[–0.13; 0.79]
Oran	0.27	0.78	[0.53; 1.02]
Ouargla	0.14	0.27	[0.07; 0.47]
Oum el bouaghi	0.36	0.89	[0.52; 1.27]
Relizane	0.12	0.3	[0.11; 0.50]
Saida	0.09	0.16	[–0.02; 0.35]
Setif	0.95	2.34	[1.97; 2.72]
Sidi bel abbes	0.03	0.05	[–0.02; 0.11]
Skikda	0.15	0.49	[0.23; 0.74]
Souk ahras	0.57	1.56	[0.95; 2.17]
Tamanrasset	0.98	1.85	[0.94; 2.75]
Tebessa	0.19	0.4	[0.17; 0.63]
Tiaret	0.07	0.17	[0.03; 0.30]
Tindouf	0	0	
Tipaza	0.43	1.2	[0.74; 1.66]
Tissemsilt	0.71	1.65	[0.96; 2.33]
Tizi ouzou	0.8	2.63	[2.13; 3.14]
Tlemcen	0.05	0.14	[0.02; 0.27]
Total	0.45		

† average annual incidence rate of visceral leishmaniasis per 100,000 inhabitants per region.

**Figure 5:**
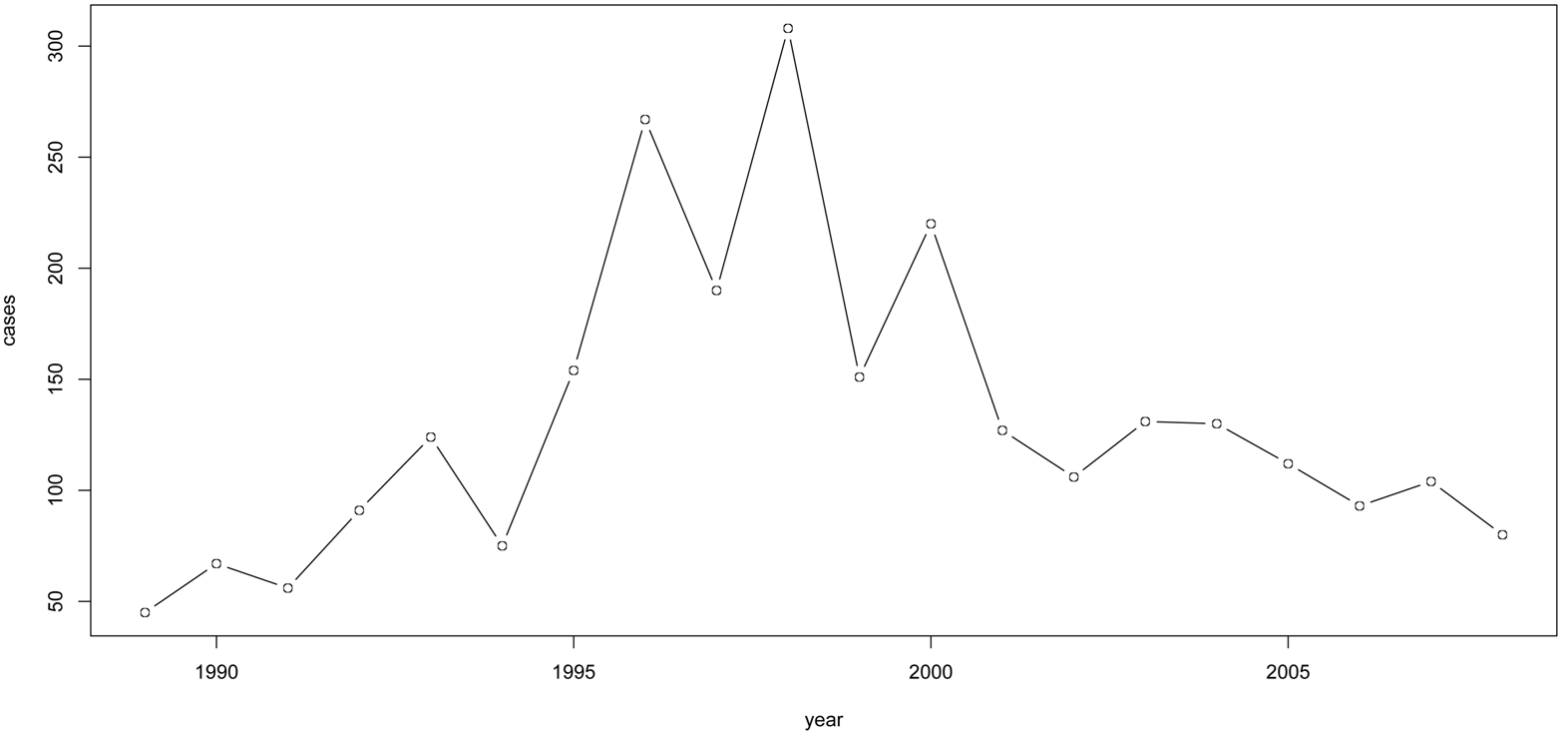
Evolution of the annual incidence of visceral leishmaniasis in Algeria (1989–2008) (N = 2631).

**Figure 6:**
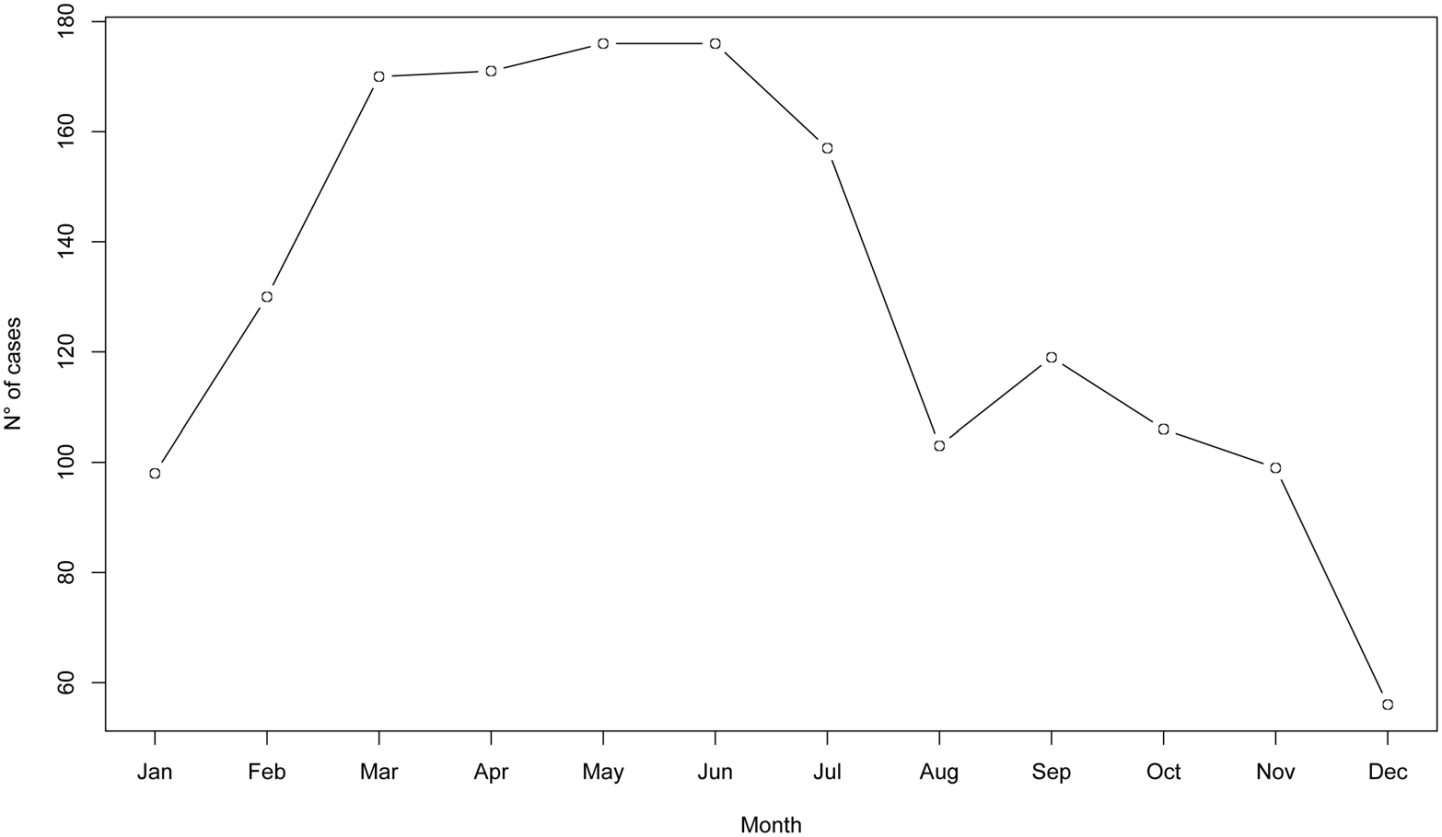
Visceral leishmaniasis distribution per month (1998–2008) (N = 1562).

The highest number of cases was reported in 1998 (308) (Figure 5). For the period 1998–2008, a clear trend towards reduction in the annual number of VL cases over time (years) was observed (Chi2 = 245.156, df = 10, p
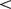
0.001). The monthly distribution of cases based on the admission date, is shown in Figure 6. Cases were reported in all months, with a minimum in December (56 cases) and a maximum in May and June (176 cases). The main cluster of cases was observed in the March–July period (850/1560 cases, 54.45%).

During our survey at the five university hospitals of Algiers we recorded 170 patients. Thirty three provinces of Algeria were represented and the majority originated from the central part of northern Algeria (124, 72.94%). Cross-checking of the data showed that only 8.24% (14/170) of the cases where notified in the INSP database.

**Figure 7:**
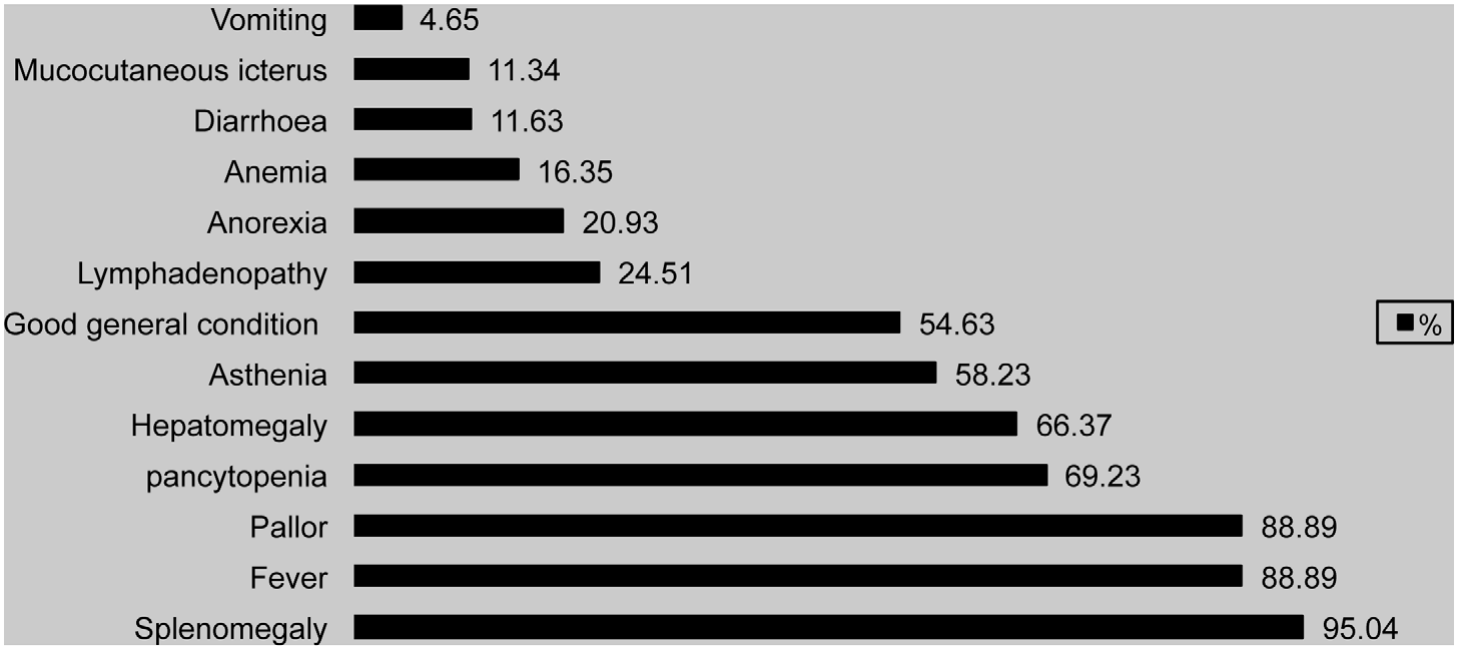
Percentage of clinical and laboratory features of visceral leishmaniasis cases in Algeria.

At admission, 56.6% of the patients reported the onset of symptoms in the previous two months, and for 41.51% of them onset did not precede 30 days. Nevertheless, in 30.19% of the cases, this value was higher than 3 months. Table 3 shows the time between symptom onset and admission. The main clinical and laboratory features of VL in Algeria are summarized in Figure 7. The most frequent findings were splenomegaly (95.04%), fever (88.89%), pallor (88.89%), and pancytopenia (69.23%). First-line VL diagnosis was based on a direct microscopic visualisation of *Leishmania* amastigotes in bone marrow aspirates (94%) and in 54.43% of cases the immunofluorescence antibody test (IFAT) was carried out. The Western-Blot technique was used for 4 patients (2.35%). Meglumine antimoniate was the first-line therapy for 94.95% of the pæ diatric VL cases. Unresponsive patients were treated with miltefosine or liposomal amphotericin B. Conventional amphotericin B was the first-line regimen for adult patients. Nine percent of patients did not respond or died during treatment.

**Table 3:** Time between symptom onset and hospitalization for visceral leishmaniasis suspicion in Algeria.

Period	Percentage	95%CI
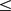 15 days	16.98	9.14–24.82
[15 30] days	24.53	15.10–33.96
[1 2] months	15.09	7.70–22.48
[2 3] months	13.21	6.29–20.13
[3 4] months	8.49	2.94–14.04
[4 6] months	8.49	2.94–14.04
[6 12] months	8.49	2.94–14.04
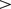 12 months	4.72	0.58–8.86

## Discussion

In the study period (1998–2008) the reported annual incidence rate of VL in Algeria decreased below 0.5 per 100,000 inhabitants. This annual incidence is comparable to what is observed in southern European countries endemic for VL [17]. This rate is however, lower than in Tunisia (1.04 per 100,000 inhabitants) (Aoun et al., 2009) and Turkey (1.6–8.53 per 100,000 inhabitants) [17]. Such incidence data should be handled with caution, as they depend much on the degree of underreporting which may be variable across countries. In our study, less than 1 in 10 cases observed in the Algiers university hospitals was actually notified to the national epidemiological surveillance centre that is the source for the official data: this tenfold under-reporting stands in sharp contrast with the mild under-reporting, referred to in [14]. Noteworthy, a relatively higher number of VL cases was observed from 1995 till 2000 (Figure 5) probably because of the political instability during the nineties that did not allow the implementation of vector control programs. The sex ratio (male:female) equals 1.24 and this is lower than that reported by [18]. It is however difficult to comment on this difference, given the small sample size of the latter study and the possibility that it is not representative of the national population.

VL cases are observed year-round in Algeria. The fact that 54.45% of all cases were reported between March to July can be explained because of the seasonal transmission of *Leishmania infantum* from May to October [8], the long incubation period of VL and the variability of individual susceptibility to the infection [19]. This is in agreement with the findings by Aoun et al. 2009 [20] in Tunisia.

From 1998 to 2008, 45 provinces on the 48 reported at least one case of visceral leishmaniasis. Djelfa, El Bayadh and Tindouf appear to be free from the disease since 1998. Geographically, VL remains a disease of the humid and sub-humid regions in the North of Algeria [6]. The number of VL cases is equally distributed over the east (40.97%) and the centre (40.59%) of northern Algeria. 72.55% of the cases in the Central region occurred in the region of la Grande-Kabylie. Nonetheless the number of cases in Tizi-Ouzou, the biggest city of la Grande-Kabylie, decreased from 285 cases (years 1985–1990) [8] to 106 cases (years 1998–2008). This drop can be explained by the awareness of the local authorities that recognized there was a VL outbreak and responded with enhanced vector control by pyrethroid spraying (Deltamethrin).

In this region in addition to dogs, wild canids, such as jackals (*Canis aureus*), could be potential feral reservoirs for *L. infantum* [21]. In fact, looking at the distribution of the jackal in Algeria [22], a concordance can be observed with the distribution of VL cases. In Bejaia, Tizi-Ouzou and Bouira, a close contact was noted between dogs and jackals. In Tizi-Ouzou, the strain *Leishmania infantum* Mon1 was isolated on a three years old jackal, captured during 2007 [23].

VL has spread southward as shown by the map (11.85% of the cases); 115 cases were reported in Biskra during this period studied versus 31 cases in the previous study from 1985 to 1990 [8]. Biskra, the gate to the great south, is rich of palm plantations. In this city, the sewage water is collected in septic tanks which today rises to the surface and pollutes the ground water. These man-made environmental changes may increase human exposure to the sandfly vector.

Moreover, cases were notified from the province of Illizi for the first time. Furthermore, when comparing between-province incidence rates, Biskra and Illizi, two provinces of the South, were respectively the third and forth most infected towns.

The survey at the five University Hospitals (UH) of Algiers documented 170 cases including one never notified pæ diatric case from Djelfa in 2008. The 30 VL cases among residents of Algiers were either persons living in the periurban areas, i.e. an agricultural area with active transmission or persons who had sojourned in other regions as Tizi-Ouzou, Bejaia or Setif. For 114 out of 170 patients the area of residence was recorded and socio-economic status only for 40. Thus, 94/114 patients were from a rural area (82.46%) and 33/40 (82.5%) had a very low social socio-economic status and lived in poor housing conditions known to be risk factors for VL [10], [24], [25].

VL remains mainly a paediatric disease in Algeria as in other countries such as Tunisia [20], Morocco [25], Albania [26] and Brazil [27]. Adult cases represent only 4.9% of the total number reported. This proportion is similar to the one in Tunisia [20] but it is different from the situation in some northern Mediterranean countries that report larger proportions of adults affected (40–70%) due to HIV-coinfection [28] and intravenous drug use. Nevertheless, a clear decrease in the incidence of Leishmania-HIV coinfection was observed by the end of the 1990s due to the routine use of highly active antiretroviral therapy in AIDS patients in most of southern Europe [29]. Thus, France reported from 1999 to 2012, 366 VL cases of which 31.4% were HIV infected, 9.6% had an immunosuppressive treatment and 3.3% had received an organ or bone marrow transplant [30]. In Italy, 14% of total VL cases notified during a 30 years period were HIV co-infected patients (1982–2012) whereas the other concomitant conditions were organ transplantation, hepatitis and liver cirrhosis, pregnancy, leukaemia, lymphoma, hepatitis B and systemic lupus erythematosus and diabetes [31]. In Greece, 15% of the VL cases were immunocompromised [32]. In our series 17 of the 25 adult VL cases observed in the hospital survey were HIV-coinfected. The eight remaining patients presented either medullary granulomatosis, diabetes, hepatitis B with tuberculosis, systemic lupus erythematosus (2 cases), leukaemia, vitamin B12 deficiency or a flu-like syndrome.

The median time lag between symptom onset and the hospitalization for suspicion of VL was in 56.6% of the cases between 0 and 2 months of which 41.51% did not exceed 30 days. These results are similar to the diagnostic delay observed in Tunisia [20] and in Italy [33]. Nevertheless, in 30.19% of the cases, diagnostic delay exceeded 3 months. This could be due to misperception of the seriousness of early signs by the child’s parents [34] or by doctors.

Until the early 1990s, pentavalent antimony was the only drug employed for the treatment of VL in the Mediterranean, with reported cure rates exceeding 95% in immunocompetent patients [35]. While conventional amphotericin B (Fungizone) is the first-line therapy for adult patients, meglumine antimoniate (Sb(v)) (Glucantime) till date remains the main treatment of paediatric VL in Algeria [28] and is available free of charge at public hospitals. Resistance to Sb(v) does not seem to be yet a major problem yet. Unresponsive patients are treated with miltefosine or liposomal amphotericin B (Ambisome) when available. The high cost of the latter did not allow its use as a first-line drug as it is the case in France since 1994, and in Italy and Cyprus [35], [36]. Recently the World Health Organization obtained a preferential pricing scheme for liposomal amphotericin B of US$ 20.00 per vial, valid for public sector agencies of developing countries for the treatment of VL [37], [38]. This scheme opens the prospect of inclusion of this highly efficacious and safe drug in therapeutic regimens.

In conclusion, VL in Algeria shows the epidemiological profile of a paediatric disease. A decrease of the annual incidence rate was observed compared to the previous decade. However, vigilance is required because of huge underreporting and an apparent propagation towards the south. Immunocompromised adults constitute another sub-population at risk. Further studies should be carried out in the most affected cities with special consideration for the spatial variability in disease occurrence.
